# Original Communications and Other Contributions

**Published:** 1881-01

**Authors:** 


Original Communications and other Contributions—Such as original papers on various subjects, and translations of interesting articles from foreign journals, etc., are respectfully and earnestly solicited from every quarter? There are many practical facts in the possession of physicians throughout the country, and particularly among those in rural districts, which *ought* to be placed upon record for the benefit of the profession generally. And we would respectfully and earnestly ask that this journal be made a medium for their promulgation, for the good of humanity, in the future. Unless these facts and experiences are recorded, they will be lost to the world in a few years, by the death or retiring of their owners from the profession, which, we insist, should not be permitted to occur. Every one who enters the profession should feel in duty bound to contribute his experience and observations, for the good of all, and not be satisfied until he knows the profession is the richer because of his having been a member thereof. We would, therefore, say to the busy, and often over-worked country practitioners, who have no time to write lengthy and ornate papers, that we will be glad to receive the merest *outlines* of their discoveries, and will write them up for the pages of the journal. Brief articles are always welcome, and we trust they will come in abundantly during the year. All communications intended for publication, books, etc., except dental, should be addressed to Dr. H. L. Byrd, the Senior Editor, at 225 N. Gilmor Street; all dental communications, &c., advertisements and other matters appertaining to the business of the journal, to the Junior Editor, Dr. B. M. Wilkerson, 68 N. Charles Street.

